# Exploring the Efficacy of Aboriginal Men’s Socioemotional Healing Programs in Australia: A Scoping Review of Evaluated Programs

**DOI:** 10.3390/ijerph22010088

**Published:** 2025-01-10

**Authors:** Elizabeth Horak, Sandra C. Thompson

**Affiliations:** 1 School of Health, Georgetown University, Washington, DC 20057, USA; 2 Western Australia Centre for Rural Health, School of Allied Health, University of Western Australia, Geraldton 6530, Australia; sandra.thompson@uwa.edu.au

**Keywords:** Aboriginal, Australia, evaluations, healing, socioemotional wellbeing, programs, family violence, cultural understanding, gender

## Abstract

Aboriginal and Torres Strait Islander (hereafter, respectfully, Indigenous) men’s health and social indicators reflect an ongoing legacy of social disruption with profound implications for broader family and community contexts. In response to recognized needs, healing programs have been implemented within Australia. The literature on relevant best practices for Indigenous men’s healing was explored to inform the planning and implementation of a local program. A scoping review of electronic databases was undertaken to retrieve information between 2012 and 2022 on social and emotional healing programs for Indigenous men that included a program evaluation. Of the 2123 identified articles, many lacked a program evaluation or were not specific to male participants, with nine meeting the inclusion criteria for the review. Six central elements that supported the programs’ reported efficacy were identified: kinship, cultural understanding, a view of healing as being holistic, a strengths-based approach, a male leadership team, and a consistent meeting space. These elements were important for the social and emotional healing of the Indigenous male participants. Based on these findings, there is an increased need for the identified elements to be incorporated into programs for Indigenous men to accompany ongoing efforts in improving the wellbeing of the Indigenous population overall.

## 1. Introduction

### 1.1. Context of the Role of Indigenous Men

The Aboriginal and Torres Strait Islander population of Australia constitutes roughly 3.3% of the population [1]. Indigenous culture centers around an extensive family network, with families having their own unique customs, ceremonies, and traditions [2].

In the early 19th century, these communities faced forceful European colonization, which brought epidemic disease and occupancy of Indigenous land [3]. Colonization also led to policies such as racial assimilation and the removal of Aboriginal children from their families, with as many as one in ten being removed [4]. This affected the entire family unit and had a marked effect on Aboriginal men. 

Traditionally, the male role in Indigenous culture has been defined through loyalty, pride, hard work, and the sharing of culture [5]. These positive attributes have been historically passed down through Indigenous male Elders to younger men. However, as their children were forcibly removed, Indigenous men saw their role as cultural knowledge sharers disappear, along with their strong relationships within the family unit and community [5]. Today, male role models are still less visible for Indigenous men than for Indigenous women throughout Australia [5].

The expectation of Indigenous males to be teachers of cultural knowledge and strong role models has had an impact on their parenting role. Prior to becoming a father, Indigenous men often report feeling unprepared and anxious yet face the cultural expectation of concealing their emotional vulnerabilities [6]. This dissonance places strain on the family unit [7]. However, a positive association between the Indigenous man’s role as a parent and their wellbeing also exists [4]. Fathers, uncles, and grandfathers are expected to teach Aboriginal children about their culture, which in turn, allows them to solidify their own cultural identity and belonging. Indigenous men have increasingly reported that taking responsibility and acting as a strong role model for their families and communities is a form of “manning up” [5].

### 1.2. Family and Domestic Violence 

Family and domestic violence (FDV) prevalence is greater in Indigenous communities than in the non-Indigenous Australian population. In 2017, most of the reported Indigenous assault cases in Australia were related to FDV, ranging from 64 to 74% of total cases, depending on the state or territory [8]. Between 2022-2023, Indigenous women were 34 times more likely to be hospitalized because of an FDV incident than non-Indigenous women in Australia [8]. Family violence perpetrated by men is associated with colonization, rigid gender norms, mental health history, and substance abuse [8]. These challenges increase the risk of FDV, which contributes to a greater burden of disease for Indigenous women aged 18–44 years than any other risk factor [9].

### 1.3. Substance Abuse 

High stress and intergenerational trauma are associated with substance abuse, a significant problem in Indigenous populations. Alcohol and drug use is higher in the Indigenous population, as they are 1.5 times more likely to drink at high levels than their non-Indigenous counterparts. The increased rate of substance abuse increases the chance of risky behavior-taking [10]. Indigenous men are also more likely than women to abuse drugs and alcohol [11].

### 1.4. Aboriginal and Torres Strait Islander Healing 

These challenges have a considerable impact on Indigenous people’s health. This population’s health is based on a culturally holistic perspective [1] encompassing a mental and physical approach to health, as well as a connection to country, spirituality, and ancestry [12]. For example, the Pitjantjara people use the word “Kanyini” to define social and emotional wellbeing, which includes aspects of creation, dreaming, soul, family, kinship, and land; as a result, they view the process of improving health as socioemotional healing emphasizing reconnection to community and family, country, culture, and self-identity [13]. Although there is not a single comprehensive definition of healing given its range of cultural interpretations, the Healing Foundation defines it as addressing “mental, physical, emotional, and spiritual needs and involves connections to culture, family, and land” [14]. As a result of the cultural prioritization of social and emotional healing, Aboriginal people recognize broader social issues as having a more significant impact on health [15].

### 1.5. Indigenous Healing Programs 

There is a clear need for the existence of programs focused specifically on holistic healing for Indigenous men [16], with the Healing Foundation established to prioritize this [14]. For Indigenous men specifically, it is pivotal for them to have programs in which they feel comfortable participating in the healing process so their underlying risk factors may be properly addressed [6].

Many programs have focused on the healing of Indigenous people across Australia, which have been compiled within several scoping and systematic reviews. There are, however, several gaps within the published literature; many reviews do not focus on programs for Indigenous men specifically, and only include general Indigenous healing programs [15,17,18,19]. Much of the previous literature also reports on programs that occurred 20–30 years ago [15,17,18,19]. Evidence from more recent healing programs is pivotal to adequately address contemporary needs and challenges. Finally, most reviews report on program descriptions and broad opinions about the program, rather than programs subject to careful evaluation [15,17,18,19,20]. Program evaluations are essential in providing evidence of program efficacy and future recommendations. Australia’s National Research Organization for Women’s Safety (ANROWS) stated that “more targeted research is urgently required to provide evidence and evaluation for effectiveness to improve the individual and service accountability outcomes”. Their report notes that this type of research focus may “provide better evidence of how to manage and rehabilitate perpetrators of family violence successfully within justice systems and communities, to ensure that Aboriginal women and children are no longer exposed to the extraordinarily high levels of violence currently being experienced” [9].

Our preliminary assessment of the literature identified a gap in research, emphasizing the need for a focused assessment of recent program strategies specifically centered on Indigenous men’s social and emotional healing and their evaluated outcomes. This review seeks to address this gap in exploring the design and impact these programs had, utilizing the research question, “What evidence has been collected from evaluations of social and emotional healing programs for Indigenous men in Australia over the past ten years to inform healing program delivery?”. Findings will inform future efforts to develop and implement social and emotional healing programs for Indigenous men in Australia.

## 2. Methods and Data

### 2.1. Study Design

A scoping review seeks to identify and map available evidence in a given field and inspire further research in its identification of current needs and gaps [21]. This scoping review maps relevant peer-reviewed and gray literature of evaluated social and emotional healing programs for Indigenous Australian men.

### 2.2. Search Strategy

An electronic database search was conducted from September 2022 to October 2022 utilizing PubMed, Australian Indigenous HealthInfoNet, PsycInfo, and SAGE Journals. In addition, Google Scholar and websites of organizations operating programs were used to identify gray literature which included relevant reports and evaluations. The key search terms used and searched for within the title, abstract, and keywords of the resources were the following: Aborigin* OR Indigen* OR “Torres Strait”; AND men OR males OR boys; AND Australia AND program OR plan OR project OR initiative; AND healing OR wellbeing OR well-being OR social OR emotion* OR “mental health” OR cultur*. Both peer-reviewed resources and the gray literature were eligible for inclusion. The gray literature is not generally as rigorously reviewed as peer-reviewed resources posing a potential issue for its quality of information and reliability. However, the incorporation of the gray literature in this review enabled a more inclusive approach to relevant evaluations of Indigenous programs.

### 2.3. Selection Criteria

The search was informed by the following eligibility criteria: open-access full-text articles written in the English language published between 2012 and 2022, undertaken in Australia, focused on Indigenous male populations, and which included a formal evaluation of a social and emotional healing program undertaken between 2012–2022. Quantitative, qualitative, and mixed-methods literature was included. Exclusion criteria were residential programs and any source that did not meet the inclusion criteria. 

**Figure 1:**
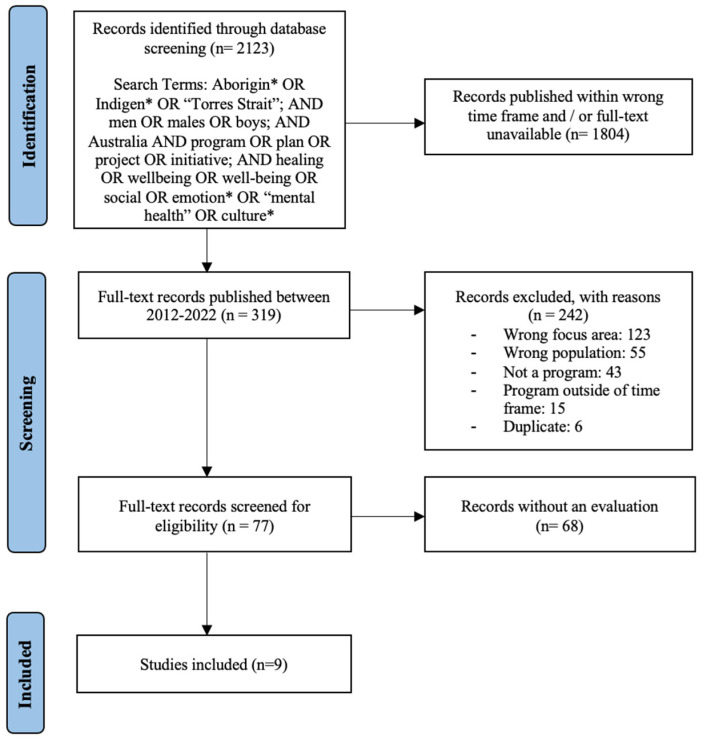
Flow diagram of the selection process for inclusion of the relevant literature.

The search was conducted in four stages based on the eligibility criteria (Figure 1).

1. Articles were initially identified using the key search terms. A librarian was consulted for the selection of these search terms and electronic databases.
2. Articles were then screened by keywords, title, and abstract. This process was done primarily by one reviewer (E.H.), with extensive consultation from the second reviewer (S.C.T.). The articles were discussed extensively by both reviewers based on the inclusion and exclusion criteria throughout the study selection process. Articles were removed if deemed irrelevant (e.g., physical healing) and/or they did not meet the eligibility criteria.
3. Articles were excluded after their full text was reviewed and deemed ineligible for inclusion. Reasons for exclusion included articles that were duplicates, not centered around a program/programs, not focused on social and emotional healing, and/or if the program was delivered to a population other than Indigenous Australian men.
4. Articles were then excluded if they did not include an evaluation or the evaluation was undertaken outside of the set time frame of 2012–2022.

## 3. Results

Based on the search criteria, nine records were included for data extraction and review. Variables from the nine studies are summarized in Table 1, detailing the design, sample size, central aim, evaluation design, key outcomes, and recommendations of each of the twelve programs.

### 3.1. Program Design 

The primary focus of each of the programs was on Indigenous men’s social and emotional healing to improve their wellbeing. This goal was structured into each of the programs, as they prioritized providing a culturally safe space for men to strengthen their wellbeing, connection with their family and culture, and engagement with the community.

The programs occurred in a variety of places across Australia: five of the six states (three programs took place in Victoria, two in Western Australia, one in South Australia, one in Queensland, one in New South Wales, and one was not specified) and one of the two territories (three programs in the Northern Territory). The twelve programs described spanned a range of settings: one urban, two regional, and three rural, and six evaluations included a combination of environments (two in both regional and urban; three in regional and rural; and one in urban, regional, and rural areas). The type of setting did not play a major role in the delivery or key outcomes of the programs, other than those that took place within the rural settings. In remote communities, group trauma is often more widespread, reducing the potential for positive interactions between men [22]. Additionally, there were often fewer resources available for social and emotional healing programs, and greater fluctuation when these resources were accessible. This was observed within the “Our Men Our Healing” initiative, which took place in rural Aboriginal communities in the Northern Territory. Even though the initiative produced many positive outcomes, one pivotal component from its evaluation was the need for increased resources in pilot communities, including support for the male program leaders [23]. This recommendation was also seen in the SMS4dads program, which was run in both regional and rural settings. The evaluation suggested the prioritization of capacity-building in these settings to reach a higher proportion of Aboriginal people [24]. The LifeCycle Youth Connect Program, run in both regional and rural settings, found participant attendance was more consistent in the Aboriginal rural communities than the regional settings despite disparities in capacity [25]. As expressed in their evaluations, these communities were very receptive to programs that targeted Indigenous male healing, further strengthening the recommendation of building capacity in these types of environments.

**Table 1:** Summary of relevant Indigenous men’s social and emotional healing programs that took place between 2012 and 2022 in Australia.

Program Name	Program Aim	Participants	Program Design	Evaluation Design	Key Outcomes	Recommendations/Needs
Men’s Sheds [26]	Offer a safe space for men to socialize and participate in health promotion, informal learning, and engage in meaningful tasks individually and as a community	61 men (Indigenous leaders, shed coordinators, and participants)	Urban, regional, and remote Australia 5 yarning circles held at the Men’s Sheds	Qualitative: semi-structured interviews	Effective development of social relations through men’s programs that provide a culturally safe space contributes to the improvement of social and physical wellbeing	Desire to run more social and emotional programs as well as programs that target both mental and physical healing Policies to prioritize social requests and contributions of Aboriginal men’s ideas
His Tribe [27]	Strengthen mental health, social and emotional wellbeing, community connection, and to reduce psychological distress	26 men completed assessments at pre—and post-program completion, and 17 and 10, respectively, participated in yarning circles at the 6-month follow up	Metropolitan Melbourne Weekly 2.5 hour evening group session at a local Aboriginal community-controlled organization over 12 weeks Cultural weekend activities	Mixed methods: Aboriginal Resilience and Recovery Questionnaire Kessler Psychological Distress Scale (K10)	Significant increase in participants’ access to personal strengths and resources, relationship–community–cultural strengths and resources, and decreases in psychological distress, which were associated with small to moderate effects that were maintained at the 6-month follow up. Lower post-traumatic stress and depression symptom severity, and higher levels of empowerment	Assess prior mental health vulnerabilities of participants Selection bias in finding participants through social media—find a more representative sample for greater statistical significance
Men’s Healing and Behavior Change [28]	Address the drivers for violence by strengthening cultural connections, develop pride and confidence, and plan a future with healthy relationships in families and communities	80 clients across programs—participants as well as administrators	Melbourne, multiple sites across Victoria Weekly men’s group sessions in Melbourne, fortnightly men’s group sessions across Victoria over the year	Mixed methods: program outcomes measured through proprietary outcomes tool used by Dardi Munwurro, cost–benefit analysis measured through a cohort approach and sensitivity testing	Both Deloitte & Healing Foundation Evaluated Programs: Greater connection to culture and stronger sense of identity Feelings of improved relationships, taking responsibility for behavior, and connections to community Decrease in alcohol and other drug usage from 80% to 34% Homelessness reduced by 100% Increase in employment Each dollar invested into Dardi Munwurro was estimated to provide a return on investment of 50–190%, with the largest return being from a decreased rate in incarceration Specific: 80% reduction in a reported recent family and/or domestic violence incident	Widen the scope of the data through recording longitudinal data on participant outcomes after program completion
Journeys Program [28]	Support the transition into healthy adulthood by providing positive support networks and building on individual resilience and knowledge, culture, emotional intelligence, health (physical and social and emotional wellbeing), relationships, responsibility, and spirit	80 clients across programs—participants as well as administrators (participants ages 10–17)	Melbourne, multiple sites across Victoria Peer group providing access to positive male mentors	Mixed methods: program outcomes measured through proprietary outcomes tool used by Dardi Munwurro, cost–benefit analysis measured through a cohort approach and sensitivity testing	Both Deloitte & Healing Foundation Evaluated Programs: Greater connection to culture and stronger sense of identity Feelings of improved relationships, taking responsibility for behavior, and connections to community Decrease in alcohol and other drug usage from 80% to 34% Homelessness reduced by 100% Increase in employment Each dollar invested into Dardi Munwurro was estimated to provide a return on investment of 50–190%, with the largest return being from a decreased rate in incarceration Specific: Number of clients engaged in some form of education almost doubled Levels of “spirit” and “culture” increased by ~5 on a 10 point scale between pre and post program reports	Widen the scope of the data through recording longitudinal data on participant outcomes after program completion
Our Men Our Healing—Tiwi Men’s Healing Program [23]	Strengthen men in heart, body, mind, spirit, and culture Increase safety for family and community Strengthen community connections between different programs and employment services	50 community members in total	Wurrumiyanga, Northern Territory Counseling, group programs and yarning circles, events/celebrations, cultural camps offered	Qualitative: interviews with participants, facilitators, service providers, and women connected to the male participants	All Our Men Our Healing Programs: Reported decrease of FDV incidence Women felt safer and more supported by male participants Increase in reported emotional wellbeing Increase in men being positive role models in communities Specific: 50% reduction in men registered in correctional services Reduction in rates of recidivism and reoffending over program course Increased male confidence	All Our Men Our Healing Programs: Young men find it difficult to connect to culture Need for increased opportunity to transfer cultural knowledge from Elders to young men Need for more resources in pilot communities and support for men leading these programs Women should be included so they are informed and can support the program
Our Men Our Healing—Gurrutu Raypirri Men’s Healing Program [23]	Strengthen men in heart/body/mind/spirit/culture Increase safety for family and community Strengthen community cultural connections	50 community members in total, men 16–60+	Maningrida, Northern Territory Art-centered activity within workshops with some weekend BBQ fishing trips	Qualitative: interviews with participants, facilitators, service providers, and women connected to the male participants	All Our Men Our Healing Programs: Reported decrease of FDV incidence Women felt safer and more supported by male participants Increase in reported emotional wellbeing Increase in men being positive role models in communities Specific: Some men found employment through selling art	All Our Men Our Healing Programs: Young men find it difficult to connect to culture Need for increased opportunity to transfer cultural knowledge from Elders to young men Need for more resources in pilot communities and support for men leading these programs Women should be included so they are informed and can support the program Specific: No place to meet posed a challenge to maintain momentum and sustainability Disperse leadership away from one man and more towards the overseeing organization (Malabam Health Board)
Our Men Our Healing—Ngukurr Men’s Cultural Healing Program [23]	Strengthen men socially, emotionally, mentally, physically, spiritually, and culturally and encourage them to be positive leaders Encourage men to pass on cultural knowledge to community	50 community members in total	Ngukurr, Northern Territory Safe space that offers counseling, social and emotional wellbeing focus and advocacy, cultural camps, and other events for men	Qualitative: interviews with participants, facilitators, service providers, and women connected to the male participants	All Our Men Our Healing Programs: Reported decrease of FDV incidence Women felt safer and more supported by male participants Increase in reported emotional wellbeing Increase in men being positive role models in communities	All Our Men Our Healing Programs: Young men find it difficult to connect to culture Need for increased opportunity to transfer cultural knowledge from Elders to young men Need for more resources in pilot communities and support for men leading these programs Women should be included so they are informed and can support the program
SMS4dads [24]	Test the acceptability and feasibility of a website offering tailored support and information to young Aboriginal fathers Adapt and test a mobile phone-based text-messaging and mood-tracker program that provided ongoing social and emotional support to fathers	20 young Aboriginal fathers	1 regional and 2 rural Aboriginal communities in New South Wales Yarning sessions “Stayin on Track”—25 texts sent over the course of 6 weeks; 5 messages sent included relevant websites Mood Tracker—4–10 messages to each father over the course of 6 weeks	Qualitative: participatory design approach, Aboriginal men as co-investigators, yarning sessions, and filming of responses about fatherhood, answers to program messages and Mood Tracker analyzed	Provided opportunities to network with other fathers and to discuss their common issues Development of participants as mentors for other young men in the community, increasing program sustainability Preliminary support for the feasibility of providing support to young Aboriginal fathers through mobile phone-based text-messaging and mood-tracking programs to assist them in the transition to fatherhood. Specifically, high level of engagement with the mood tracker messages, suggesting it could be an appropriate tool for monitoring of emotional health and coping	Need to evaluate the number of views per video on the website, and the number of clicks on the links to other websites when Aboriginal fathering websites presented Need for a greater range of young fathers to be involved in reviewing and evaluating the efficacy of the text messages Recommendation to give greater attention to capacity building, empowerment, and community ownership
Strong Fathers and Strong Families [4]	Promote men’s wellbeing by emphasizing the value of their role as proud Aboriginal and Torres Strait Islander family members and healthy role models for their children	25 Aboriginal and 6 non-Aboriginal stakeholders	Lower Gulf of Carpentaria Weekly 3-h sessions over 6 weeks	Qualitative: yarning sessions, data collected using an audio recorder	Highlighted key challenges and opinions Aboriginal men have about strengths and limits to their fathering abilities including female bias in the health system, shame, lack of confidence, and disempowerment Effectiveness of men’s groups for young fathers expressed by participants	Need for increased awareness in services for men’s parenting role Health worker involvement in yarning sessions Increase in local media involvement with promotion of the parenting role and responsibilities of fathers, uncles, and grandfathers
Violence Prevention Program [29]	Decrease reoffending patterns for Aboriginal men through working to improve the participants’ social and emotional wellbeing	92 men who participated in the program between 2014–2016, 81 matched to untreated person in comparison pool Comparison sample—157 incarcerated men who were released during the same 3-year period	Prisons in South Australia Weekly group sessions over 9 months	Mixed-methods: Intention-to-treat research design from completers and non-completers of the program, cost–benefit analysis	65% reduction in the likelihood of violent reoffending for Aboriginal men, which had a significant association with participation in the program No difference in number of days to recontact correctional services after program participation Reincarceration cost in terms of prison bed days is about AUD 1.9 million less than those incurred by the “no treatment” group Taxpayer costs were AUD 172,221 less than costs of the “no treatment” group For every taxpayer dollar spent on the treatment intervention a AUD 1.13 benefit was returned over a 3.8-year observation time frame	Continued work needed to establish when, how, and why violence prevention programs are effective when delivered to men in prisons
Quop Maaman: Aboriginal Fathering Project [30]	Encourage positive and healthy Aboriginal fathering based on appropriate research and consultation	10 Aboriginal men and boys	Southwest of Western Australia in and around Perth Series of 6 workshops offered over 6 months	Mixed methods: formative design, evaluating the work while it is in the process done in 3 phases	Reported learning of family connections, the difference between the past and present, Elders’ cultural knowledge, new language, and different views of fathering	More yarning and interaction Involve the Elders to a greater extent More cultural activities and awareness of different circumstances men have in their lives Uptake of the program by correction centers and schools
Lifecycle Youth Connect Program [25]	Encourage youth skill development, awareness of resources, positive relationships, growth in self-esteem, and reduction of crime and anti-social behavior	Primarily Aboriginal male youth, totaling 535 people	7 communities in Broome and Derby 5 Aboriginal communities—Bidyadanga, Ardyaloon, Djarindjin/Lombadina, Beagle Bay, Looma in Western Australia Series of 60 mobile bike repair sessions across settings run over 12 months	Mixed methods: process evaluation framework Data collected through written surveys and interviews with young people, stakeholders Informal interviews with parents and guardians Observations by an independent evaluator at 9 sessions in 6 different locations	More consistent attendance in Aboriginal communities than Broome/Derby Clear evidence of youth skill development and an increase in self-esteem Strong support for increased awareness of resources, enhancement of relationships correlated with involvement in the program Unclear data on reduction in crime/anti-social behavior because of the program (anecdotal improvements from community members and police in Aboriginal communities, but not supported in Broome/Derby)	Expand program to other locations Increased social network presence Development of an attendance plan Work to have more adults/parents at sessions Adding a structured activity to maintain interest during sessions where there is often low attendance Provide healthy food or a BBQ at the end of a session Have the Lifecycle team visit more often Option to offer motorbike repair More in-depth conversations with the youth who attend Collect and record data more systematically (age of participants, returning participants, date and involvement of project partners in sessions)

### 3.2. Evaluation Design 

The evaluations used different modes of data collection, collecting qualitative data or utilizing a mixed-methods approach. Six programs collected qualitative data using semi-structured interviews and yarning sessions with the program participants and facilitators. The “Our Men Our Healing” project conducted interviews that also included Aboriginal service providers and women connected to the male participants [23]. With the SMS4dads project, the interviews were undertaken by independent evaluators using a participatory design approach. The project recruited program participants as co-investigators who held conversations and filmed responses that centered around Aboriginal fatherhood [24].

The remaining six programs utilized a mixed-methods approach for evaluation, which included the use of a range of evaluative tools. Some programs used formal measurement scales, such as the Kessler Psychological Distress Scale (K10) used by the His Tribe program [27]. Other programs used more Aboriginal-specific tools, including the Aboriginal Resilience and Recovery Questionnaire used by His Tribe [27] and the Dardi Munwurro proprietary outcomes tool used by the Men’s Healing and Behavior Change Program and the Journeys Program [28]. These methods were used to provide a more culturally appropriate means of gathering data. 

The mixed-methods evaluations utilized different overall designs. Both the Quop Maaman: Aboriginal Fathering Project [30] and the LifeCycle Youth Connect Program [25] conducted their evaluations using a process evaluation framework with the outcomes of the programs evaluated concurrently with program delivery. For the Quop Maaman project, this led to a formative design in which the recommendations were implemented and evaluated again within a three-phase process [30]. The Violence Prevention Program was evaluated using an intention-to-treat research design, with the outcomes compared between program participants and a control sample of men who did not complete the program [29].

Three of the mixed-methods programs conducted a cost–benefit analysis. The cost–benefit analysis for the Men’s Healing and Behavior Change Program and Journeys Program was conducted using a cohort approach and sensitivity testing [28]. The Violence Prevention Program focused primarily on crime-related costs and compared their occurrence of relevant indices in participants with a matched sample of male non-participants [29].

### 3.3. Thematic Analysis

A full-text review was conducted by both reviewers to determine themes included within each article. One reviewer (E.H.) developed a table that organized program elements from each article. This table was then assessed and discussed with the second reviewer (S.C.T.). The overlap identified by both reviewers between these elements led to the establishment of key themes. Based on this, the key program elements for effective participant socioemotional healing were kinship, cultural understanding, a view of healing as being holistic, a strengths-based approach, male leadership, and a physical meeting space. Efficacy was determined through the analysis of key outcomes as determined by the program evaluations, reflected in Table 1. Table 2 displays that these central elements reoccur, reflected across all the included programs. 

### 3.4. Kinship

Kinship was a central component in all the programs, as reflected in Table 2. Kinship supported the programs’ unifying goal: to strengthen Indigenous men’s connections with other Indigenous male participants as well as their families and communities.

The implementation of yarning sessions within most programs developed connections between the participants. For example, the Men’s Sheds program centered around yarning, offering men the opportunity to share their experiences and learn from one another. As a result, they reported having positive social interactions which improved their socioemotional wellbeing [26].

Another component of kinship between participants was the establishment of mentorship. The SMS4dads program connected older fathers to younger fathers, who gave them support and advice. Due to the success of this mentorship, the evaluation of the program recommended an increased involvement of older fathers who could serve as mentors to strengthen participant kinship levels and the program’s sustainability [24].

Familial kinship was a focus area within the three programs under the “Our Men Our Healing” initiative. These program evaluations included feedback from women connected to the male program participants. With their involvement, the program facilitators were better able to understand the partners’ dynamics and potential of FDV prevalence. As a result of their inclusion, the women reported feeling a greater sense of safety and support from the male participants, as well as an increased understanding of the program. Their involvement also enhanced the validity of the evaluations in offering external feedback. As a result, the three evaluations recommended the inclusion of relevant women in future evaluations [23].

The Our Men Our Healing initiative additionally had a focus on developing strong community role models, which resulted in the increased positive engagement of male participants in this position [23]. Ultimately, the initiative’s focus on kinship led to an increase in positive, healthy engagement and created a more supportive environment for Aboriginal men to facilitate their healing. 

### 3.5. Cultural Understanding 

All of the program evaluations stressed the importance of providing a culturally competent approach for the Indigenous male participants (Table 2). This element was particularly emphasized through the inclusion of cultural activities. For instance, His Tribe incorporated a smoking ceremony and shared meal at the beginning of each of its meetings [27]. Other programs such as the Tiwi Men’s Healing Program included more sporadic cultural activities, like on-country camping. Even though these were less consistent, the inclusion of cultural activities within the program led to an emergence of cultural celebrations and ceremonies in one Aboriginal community, which had not occurred in decades [23].

Cultural safety was also assured through the leadership of Indigenous people. This was found within each of the included programs and directly influenced the programs’ effectiveness. For instance, with the Strong Fathers and Strong Families program, participants expressed a preference for culturally appropriate services run by Aboriginal men. This preference stemmed from Indigenous role models’ cultural understanding, which provided a more culturally adequate environment for the participants and increased participation within the program [4].

Because of the programs’ cultural understanding, male participants reported an increase in receptivity and engagement. For example, the SMS4dads program included an element entitled, “Staying on Track,” which disseminated five websites about fathering, as well as maternal and child health. The evaluators noticed that the website with the most clicks was entitled, “Routines: Aboriginal and Torres Strait Islander parents.” This was the only website that explicitly noted its Indigenous-specific perspective within the title. This revealed Indigenous fathers’ increased receptivity to accessing information when it was deemed culturally relevant and emphasized the importance of altering resources based on cultural needs [24].

Despite these efforts, cultural barriers still posed a challenge in some programs. For example, within the Our Men Our Healing programs, the younger male participants reported having difficulty connecting to culture. As a result, the program evaluation recommended Elders increase the sharing of specific cultural knowledge with younger participants in future programs [23]. Therefore, it is important for programs to focus on young Indigenous people who may feel disconnected from their culture.

### 3.6. Holistic Healing

As shown in Table 2, all programs in this review were healing programs with a holistic perspective. Indigenous people in Australia view healing as being physical, mental, and spiritual. As a result, the surveyed healing programs focused greater attention on the improvement of social and emotional wellbeing rather than physical health outcomes. For example, the Men’s Healing and Behavior Change program sought to strengthen Indigenous wellbeing by specifically addressing drivers for FDV. Due to this multifaceted goal, the program’s outcomes had widespread healing effects including a decrease in alcohol and other drug usage from 80% to 34%; an increase in employment; an 80% decrease in reported FDV; and a reduction in homelessness by 100% for all program participants [28].

The Journeys Program also emphasized holistic healing, specifically measuring spirit and culture. Participants rated both their levels of spirit and culture to have increased by 5 points (out of 10) from before program participation to after program completion. The Journeys Program viewed these elements as being intrinsic to a holistic healing approach and prioritized their measurement to better account for all aspects of Indigenous wellbeing [28].

### 3.7. Strengths-Based Approach 

All the programs took a strengths-based approach, in which they focused on participants’ already-existing positive assets to support their own healing. This method was especially seen within the two activity-based programs. 

LifeCycle Youth Connect focused on youth skill development through holding a series of bike repair sessions. In building on participants’ strengths in learning to repair bikes, they reported an increase in their self-esteem, awareness of resources, and engagement with others involved in the program [25].

The Our Men Our Healing Gurrutu Raypirri Men’s Healing Program is centered around art activities. As a result of the positive reinforcement built into the program to encourage men’s existing artistic strengths, most of the participants reported feeling increased empowerment. Some of the participants went on to find employment by selling their art. Given the program’s strengths-based approach, the evaluation emphasized the increased social and emotional healing of the participants [23].

Most of the programs were not activity-centered and were more focused on yarning sessions. Both the Aboriginal male participants and program leaders were able to build upon their own strengths and vulnerabilities by sharing their stories. With the His Tribe program, for example, the participants reported a significant increase in their personal strengths [27].

### 3.8. Male Leadership Team

The leadership team for most of the programs consisted of Indigenous males. The programs stressed the importance of male leadership, as they “promote male autonomy” [4], facilitate learning from past and present male leaders [30], and are more culturally acceptable [4]. In addition to Indigenous men being the central leaders of the programs, some of the evaluations suggested the utility of including other populations within the program facilitation. For example, the Strong Fathers and Strong Families recommended health workers be involved in the yarning sessions to gain a greater perspective on Indigenous men’s specific needs [4]. The LifeCycle Youth program evaluation called for the inclusion of parents to strengthen their connections with their child participants [25].

The male leadership team within each program was impactful, except for the Gurrutu Raypirri Men’s Healing Program. In this program, most of the leadership responsibilities were placed upon one Aboriginal Elder. This occurred because it took an extended period of time to find and allocate leadership to the Malabam Health Board, the managing organization of the program. The program evaluation reinforced the necessity for a supervising organization to facilitate these responsibilities efficiently so the program may remain effective and sustainable [23].

### 3.9. Consistent Meeting Space 

Most of the programs had a consistent meeting space in which the Aboriginal men felt comfortable participating. For instance, the Men’s Shed program operated across Australia and often met in spaces like community gardens and garages, depending on the location. This created a consistently supportive environment for participants to establish their trust and further encouraged their active involvement [26]. All the included programs had a consistent meeting space except the Gurrutu Raypirri Men’s Healing Program. This evaluation noted that the lack of a consistent meeting space posed a challenge to the maintenance of program momentum [23].

### 3.10. Specific Program Focus

Some of the programs focused more specifically on certain Indigenous male populations in need of social and emotional healing. For example, three programs (Strong Fathers and Strong Families, SMS4dads, and Quop Maaman: Aboriginal Fathering Project) emphasized the importance of social and emotional healing for Indigenous fathers. Their yarning sessions allowed the Indigenous fathers to identify key challenges and strengths and learn from other Indigenous fathers’ specific experiences. The programs also had an effective mentorship element, in which older Indigenous fathers were able to provide support, advice, and cultural knowledge to younger Aboriginal fathers. The SMS4dads program differed from the other two parenting programs in its inclusion of texts that disseminated online parenting resources as well as mood-tracking questionnaires [24]. The program evaluations recommended increased awareness of specific services for Indigenous fathers as well as media involvement in promoting the role of positive fatherhood [4,24].

Two programs (the Journeys Program and LifeCycle Youth Connect Program) were youth oriented. Both were centered on yarning sessions, although they were very different in scope. While the Journeys program was primarily focused on using conversation and mentorship to aid in the healthy transition of male Indigenous youth to adulthood [28], LifeCycle Youth Connect focused more on skill-building in hosting bike repair sessions [25]. As a result of the Journeys program’s focus, engagement with education increased, and the cost–benefit analysis found a return on investment of 190% within seven years after program completion. The largest stream of benefits from this program was derived from the predicted decrease in incarceration rates [28]. Both this program and the LifeCycle Youth Connect program were successful in facilitating in-depth conversations with the young participants, which increased levels of self-esteem, awareness of resources, and connection to culture [25,28].

## 4. Discussion

**Figure 2:**
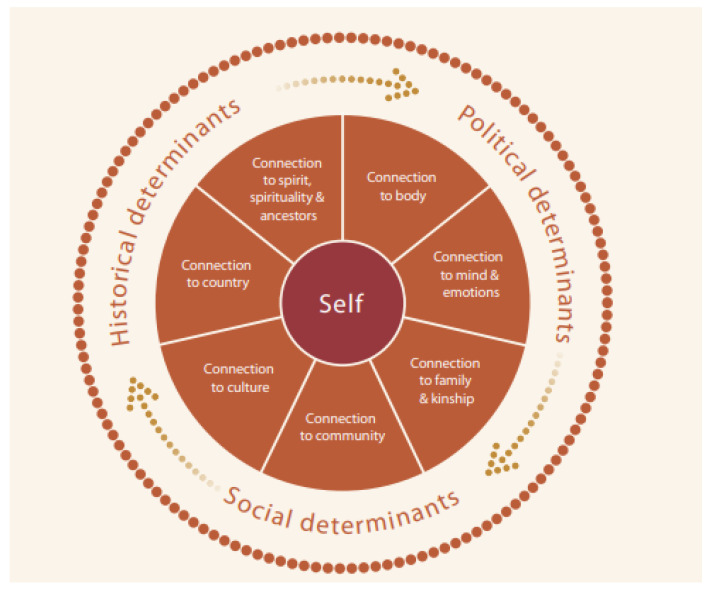
Australian Aboriginal model of social and emotional wellbeing [31].

This review provides evidence gathered from program evaluations across Australia that supports the efficacy of prioritizing kinship, cultural understanding, holistic healing, a strengths-based approach, a male leadership team, and a consistent meeting space within Indigenous men’s healing programs. It included sources that were published between 2012 and 2022 and were evaluated within that time frame. This period was chosen to focus the research on more recent Indigenous programs. The available program reviews expand on sources primarily published 20–30 years ago. Despite this gap, most of the programs included in this review reflected similar effective components to older programs including holistic healing, cultural understanding, kinship, and a strengths-based approach [17,20]. This emphasizes the consistent efficacy these elements have had in Aboriginal healing programs over time. Their importance is reflected in the National Strategic Framework for Aboriginal and Torres Strait Islander Peoples’ Mental Health and Social and Emotional Wellbeing. This framework takes a holistic healing approach, highlighted in Figure 2 [31]. The program evaluations within this review emphasize the importance of prioritizing these framework elements in programs for Indigenous men.

A consistent meeting space had not been identified in previous reviews or in Figure 2 as being an important element for Indigenous men’s healing programs. The inclusion of this element in more recent programs suggests the growing significance of providing Indigenous people with a more permanent healing environment. A consistent meeting space would establish program continuity, as well as encourage further routine Indigenous leadership. This need has also been identified through an evaluation of the Canadian Aboriginal Healing Foundation, which found healing centers to be “one of the most effective investments towards positive outcomes for those suffering from the impact of intergenerational trauma and grief,” with experts stressing their need in Australia for the improvement of Indigenous people’s social and emotional wellbeing [32].

A male leadership team was another central element that was not strongly emphasized by past evidence. This reinforces the need for increased attention to Indigenous male-specific needs, as only one past review was identified as having this focus [20]. The source noted the importance of running programs specifically for Indigenous men given their burden of intergenerational trauma related to colonization and FDV incidence. However, it did not address the need for male leadership found within this review [20]. This review identified male leadership as pivotal for providing a culturally safe space as well as empowering Indigenous men to design and lead programs, increasing receptivity and sustainability within Australia. Indigenous male leadership opportunities need to be further prioritized in order to support Indigenous men’s socioemotional healing outcomes.

### 4.1. Priority Given to Male Needs 

The findings of these male-specific programs align primarily with the Australian Aboriginal model of social and emotional wellbeing [31], while highlighting the unique inclusion of a consistent meeting space and a male leadership team. As the identified programs were all tailored to Indigenous males, many of the evaluations emphasized the importance of awareness and adequate funding of the population’s specific needs [26]. The Closing the Gap initiative has made broad strides in decreasing the disparity between Indigenous and non-Indigenous health outcomes across Australia, and the National Men’s Healthy Strategy (2020–2030) has put forth initiatives to prioritize Australian men’s specific needs. For instance, given the success of the Men’s Sheds program, the Australian Government (under the National Men’s Healthy Strategy) is set to invest millions of dollars to further develop their infrastructure and increase future sustainability [33]. This will benefit Men’s Sheds, but funding needs to be expanded to other Aboriginal men’s programs that lack capacity and sustainability.

### 4.2. Extension of Programs 

The development of additional social and emotional programs targeting holistic healing for Indigenous men has been strongly recommended [25,26]. However, this is often an issue given the system in which programs are funded and run in Australia. There is currently insufficient infrastructure and investment for sustainable program development because of a dependence on short-term funding [32]. As a result of this, the capacity and sustainability to extend the programs elsewhere to sites such as schools, healthcare facilities, or other communities is limited [25,26,30]. Additionally, healing often takes an extended period of time. Therefore, to sufficiently meet this need, long-term programs, healing centers, and other resources need to become more widely available [20].

### 4.3. Involvement of Elders

Three of the twelve included programs called for the increased participation of Aboriginal Elders to aid in the program design, spread cultural knowledge, and encourage the male participants to follow their example, becoming leaders within their communities [23,25,30]. The SMS4dads program evaluation noted a more frequent lack of engagement by younger men and connected it to the need for older Aboriginal men to act as mentors within the program [24].

### 4.4. Logistical Program Elements

Although the reported programs were successful, many of the evaluations included recommendations for how they could be improved in the future. Some evaluations recommended increased systematic data collection on participants [24,27], stating this could be done through enhanced pre-surveys to better assess participants’ mental health and wellbeing [27] and post-surveys, to gain stronger longitudinal evidence [28]. They also recommended a more significant co-design element, directly involving Indigenous men in the evaluation [24].

Many Indigenous programs are unable to conduct formal evaluations which require additional resources and expertise. Successful evaluations require financial capacity, program sustainability, participant receptivity, and a dedicated team of evaluators. Such resources may be unavailable for Indigenous programs. Therefore, these components need to be prioritized to further evaluate these programs and gather greater evidence on best practices in the field.

This research utilized full-text studies available through online searching. Often, Indigenous programs are not available online. Future research in this field could consider identifying programs that do not have published evaluations online which may be more inclusive of relevant programs and provide more evidence of the successful elements within socioemotional healing programs for Indigenous men in Australia.

## 5. Conclusions

A total of twelve evaluations done on Australian Indigenous men’s social and emotional healing programs over ten years were included in this review. The most central elements to benefit the healing of the Indigenous male participants were kinship, cultural understanding, a holistic view of healing, a strengths-based approach, a male leadership team, and a consistent meeting space.

Indigenous males in Australia have specific needs that must be addressed to promote their socioemotional healing. Healing programs that are designed to include these elements may further support Indigenous male self-determination, a strengthened connection between participants, and the sustainability of outcomes such as socioemotional healing and a reduction of structural violence. With the evidence collected within this review, policymakers may be further encouraged to support the prioritization of Indigenous men’s healing initiatives.

## Figures and Tables

**Table 2:** Inclusion of central elements related to efficacy across Indigenous men’s social and emotional healing programs.

Program	Kinship	Cultural Understanding	Holistic Healing	Strengths-Based Approach	Male Leadership Team	Consistent Meeting Space
Men’s Sheds [26]	✓	✓	✓	✓	✓	✓
His Tribe [27]	✓	✓	✓	✓	✓	✓
Men’s Healing and Behavior Change [28]	✓	✓	✓	✓	✓	✓
Journeys Program [28]	✓	✓	✓	✓	✓	✓
Our Men Our Healing—Tiwi Men’s Healing Program [23]	✓	✓	✓	✓	✓	✓
Our Men Our Healing—Gurrutu Raypirri Men’s Healing Program [23]	✓	✓	✓	✓		
Our Men Our Healing—Ngukurr Men’s Cultural Healing Program [23]	✓	✓	✓	✓	✓	✓
SMS4dads [24]	✓	✓	✓	✓	✓	✓
Strong Fathers and Strong Families [4]	✓	✓	✓	✓	✓	✓
Violence Prevention Program [29]	✓	✓	✓	✓	✓	✓
Quop Maaman: Aboriginal Fathering Project [30]	✓	✓	✓	✓	✓	✓
LifeCycle Youth Connect [25]	✓	✓	✓	✓	✓	✓

## Data Availability

No new data were created or analyzed in this study. Data sharing is not applicable to this article.
